# Systematic literature review of the impact and effectiveness of monovalent meningococcal C conjugated vaccines when used in routine immunization programs

**DOI:** 10.1186/s12889-020-09946-1

**Published:** 2020-12-09

**Authors:** Myint Tin Tin Htar, Sally Jackson, Paul Balmer, Lidia Cristina Serra, Andrew Vyse, Mary Slack, Margarita Riera-Montes, David L. Swerdlow, Jamie Findlow

**Affiliations:** 1 Medical Development, Scientific & Clinical Affairs, Pfizer, 23-25 Avenue Docteur Lannelongue, Paris, 75014 France; 2 P95 Epidemiology and Pharmacovigilance, Leuven, Belgium; 3 Medical Development, Scientific & Clinical Affairs, Pfizer, 500 Arcola Road, Collegeville, PA 19426 USA; 4 Medical Development, Scientific & Clinical Affairs, Pfizer, Surrey, UK; 5 School of Medicine, Griffith University Gold Coast campus, Southport, Queensland 4222 Australia

**Keywords:** Meningococcal disease, immunization, impact, effectiveness, herd protection

## Abstract

**Background:**

Monovalent meningococcal C conjugate vaccine (MCCV) was introduced into the routine immunization program in many countries in Europe and worldwide following the emergence of meningococcal serogroup C (MenC) in the late 1990s. This systematic literature review summarizes the immediate and long-term impact and effectiveness of the different MCCV vaccination schedules and strategies employed.

**Methods:**

We conducted a systematic literature search for peer-reviewed, scientific publications in the databases of MEDLINE (via PubMed), LILACS, and SCIELO. We included studies from countries where MCCV have been introduced in routine vaccination programs and studies providing the impact and effectiveness of MCCV published between 1st January 2001 and 31st October 2017.

**Results:**

Forty studies were included in the review; 30 studies reporting impact and 17 reporting effectiveness covering 9 countries (UK, Spain, Italy, Canada, Brazil, Australia, Belgium, Germany and the Netherlands). Following MCCV introduction, significant and immediate reduction of MenC incidence was consistently observed in vaccine eligible ages in all countries with high vaccine uptake. The reduction in non-vaccine eligible ages (especially population > 65 years) through herd protection was generally observed 3–4 years following introduction. Vaccine effectiveness (VE) was mostly assessed through screening methods and ranged from 38 to 100%. The VE was generally highest during the first year after vaccination and waned over time. The VE was better maintained in countries employing catch-up campaigns in older children and adolescents, compared to routine infant only schedules.

**Conclusions:**

MCCV were highly effective, showing a substantial and sustained decrease in MenC invasive meningococcal disease. The epidemiology of meningococcal disease is in constant transition, and some vaccination programs now include adolescents and higher valent vaccines due to the recent increase in cases caused by serogroups not covered by MCCV. Continuous monitoring of meningococcal disease is essential to understand disease evolution in the setting of different vaccination programs.

## Background

Invasive meningococcal disease (IMD), caused by *Neisseria meningitidis,* is a leading cause of bacterial meningitis and sepsis [1]. IMD is severe with high morbidity and mortality [1, 2] and is considered an important global health issue [3]. Five serogroups (A, B, C, W, and Y) of *N. meningitidis* have historically been responsible for most IMD cases, although striking regional differences exist with a highly dynamic epidemiology determining global distribution changes over time [3, 4].

An increase in serogroup C invasive meningococcal disease (IMD) due to hyperinvasive sequence type 11 (ST-11) occurred in many countries during the 1990s. As cases were occurring across all ages including infants and toddlers, the currently available plain-polysaccharide vaccines were not suitable for implementation due to poor immunogenicity in children less than 2 years of age. The development of the *Haempophilus influenzae* type b (Hib) conjugate vaccine had successfully demonstrated the benefit of conjugation in producing a vaccine immunogenic in infants. Consequently, monovalent MenC conjugate vaccines (MCCV) were developed by multiple manufacturers and these were subsequently introduced across Europe and globally [5]. Three monovalent MCCV were licensed, two vaccines used CRM-197 as carrier protein and one used Tetanus toxoid (TT), which have been followed by three quadrivalent ACWY meningococcal conjugate vaccines (MCV4), each conjugating either CRM-197 or TT or diphtheria toxoid (DT), a monovalent meningococcal A conjugate vaccine conjugating to TT and two combination meningococcal conjugate vaccines, a Hib-MenC conjugate vaccine and a Hib-MenCY conjugate vaccine, both conjugating to TT [6]. The United Kingdom (UK) was the first country to incorporate MCCV into the routine immunization schedule in 1999, closely followed by several other countries [7–11]. MCCV is part of the national immunization program (NIP) in 17 countries in Europe [12]. While these monovalent or quadrivalent meningococcal NIPs have been largely successful in reducing the incidence of MenC IMD in these countries [13], outbreaks still occur. For example, men who have sex with men have been identified as a potential high risk group in recent years following outbreaks in Canada, the United States, Germany, France and Belgium [14–18]. Implementation of MCCV NIP schedules have varied, with some countries focusing on infant vaccination with or without a booster dose, some vaccinating toddlers and some including catch up campaigns at time of implementation. Over time, schedules within some countries have been adapted in response to the evolving MenC epidemiology and to optimize protection beyond infancy through both direct and indirect (herd) protection. This systematic literature review aims to summarize the global evidence on the impact and effectiveness for routine vaccination with MCCV on MenC IMD, particularly from the point of view of the different vaccination schedules and strategies implemented as well as the duration of protection.

## Methods

We conducted a systematic literature search for peer-reviewed, scientific publications in the databases of MEDLINE (via PubMed), LILACS, and SCIELO. Search terms incorporated terms for the vaccine [((Meningococcal OR Meningit*) AND Conjugate AND (“Serogroup C” OR MCC) AND (vaccine OR vaccination))], outcome [(Hospital OR Hospitalization OR Hospitalisation OR admission OR invasive meningococcal disease OR IMD)], and effect measurements [(“Vaccine effectiveness” OR “odds ratio” OR “OR” OR “Relative risk” OR “RR” OR “Hazard ratio” OR HR OR Incidence OR rate OR trend OR epidemiology OR evolution)]. In PubMed, filters were used to include human studies only. Additional references were included after hand search on citations of the included studies (snowballing).

We included studies from countries where any MCCV was introduced in routine vaccination programs (i.e. included in the NIP), populations of all ages, single or multiple hospital settings, and observational study designs (e.g. cohort, case-control, surveillance-based, ecological) published from 1st January 2001 to 31st October 2017. We included all studies evaluating MCCV regardless of commercial brand. Our outcome of interest was hospitalization for MenC IMD, and measures of interest were Vaccine Effectiveness (VE) and impact. VE is estimated using an observational approach by comparing vaccinated and unvaccinated individuals in populations eligible to receive the vaccine and often uses a case-control or cohort study design or the screening method VE is usually calculated as 1- relative risk or odds ratio or hazard ratio and expressed as a percentage. The impact of the vaccination program is usually estimated by comparing the incidence rates of the disease in the population in the presence and absence of the program, with incidence rates before and after implementation of the program most commonly compared. The impact usually expressed as a rate reduction in incidence [19]. We included studies published in English, French, Spanish, Portuguese, Dutch, German, and Italian.

We excluded data from countries where MCCV were not part of the routine vaccination program, and studies reporting on higher valent (e.g. quadrivalent meningococcal conjugate vaccine) or combination vaccines except for studies reporting on the MenC-Hib combination vaccine in the UK and Australia, which were included. Review papers were excluded. Two reviewers (HS and MRM) screened the titles and abstracts of records identified through database searches for their relevance, based on the inclusion/exclusion criteria. In case of doubt, a third opinion was sought from a third reviewer (MTTH). The full-text review of the articles retrieved in the first screening step was conducted by a single reviewer (HS). A single reviewer performed the grey literature search (HS).

Data from the eligible full-text papers identified in the second selection step was extracted by one reviewer (SJ) using a standardized extraction form adapted from the Preferred Reporting Items for Systematic Reviews and Meta-Analyses (PRISMA) checklist. Where more than one report on the same study (population) was identified, data collected from these reports were merged into a single entry. For quality control, re-extraction of 10% of the papers was completed by a second reviewer. Data were collected on VE (i.e. VE, RR, OR, HR) and impact (IRR and incidence rate differences). Data on the population (age, size, setting, location), study design, vaccine schedule, age at vaccination and time since vaccination, case definition and diagnostic method, outcome measures and outcomes (including confidence intervals around the estimate) were collected. No formal quality assessment or evidence grading of the studies was conducted. Descriptive analysis was conducted. Meta-analysis was not considered appropriate given the different methodologies of the studies, the heterogeneity of the estimates (delay between vaccination and outcome estimation, age-group stratification, and study setting). The full protocol is available as Supplementary document 1.

## Results

**Fig. 1:**
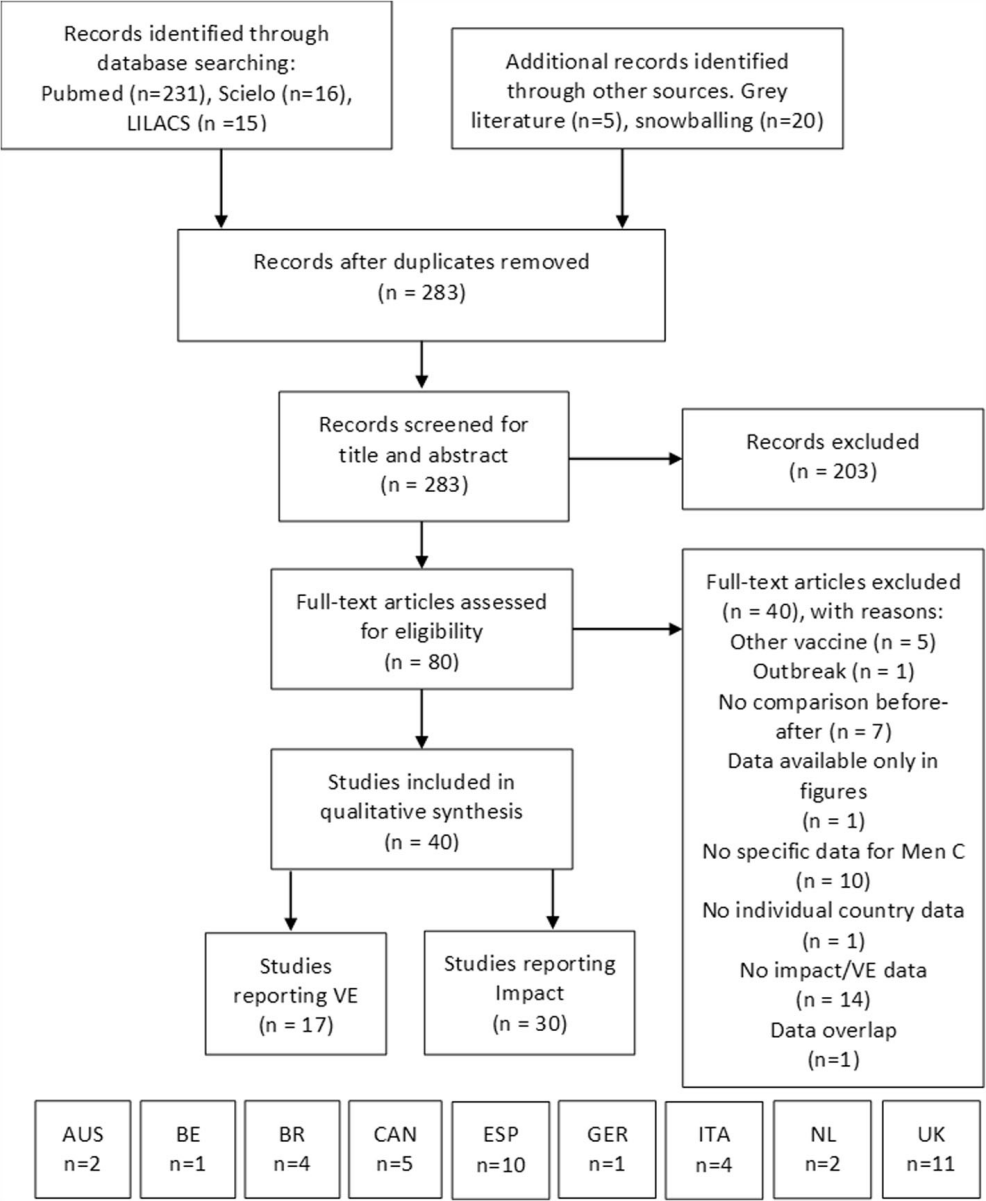
Selection flowchart of included studies. Abbreviation: AUS: Australia; BE: Belgium; BR: Brazil; CAN: Canada; ESP: Spain; GER: Germany; ITA: Italy; NL: Netherlands; UK: United Kingdom

The initial search yielded 283 articles, 203 articles were excluded after reading the titles and abstracts. 80 articles full text were read and assessed for eligibility and 40 studies were finally included (Fig. 1). A total of 30 studies reported impact of MCCV vaccination programs and 17 studies reported vaccine effectiveness (direct effect) of MCCV. Studies came from 9 different countries; 11 from England and Wales/UK [20–30], 10 from Spain [31–40], 5 from Canada [41–45], 4 each from Brazil [46–49] and Italy [50–53], 2 each from Australia [54, 55] and the Netherlands [56, 57], and one each from Germany [58] and Belgium [59]. The MCCV were introduced as part of the routine childhood NIP in all these countries. A catch-up campaign was also implemented in UK, Spain, Brazil, Belgium, Australia and the Netherlands. A list of summary study characteristics is presented in Supplementary Table 1.

### Impact of MCCV programs

A total of 30 studies in 9 countries reported the impact of MCCV NIPs on the incidence of MenC disease. Given the differences between countries in terms of prior non-conjugate vaccination program, dosing schedule, vaccination coverage, recommended ages, and the use of catch-up campaigns, we have summarized the impact per country.

#### United Kingdom

**Fig. 2:**
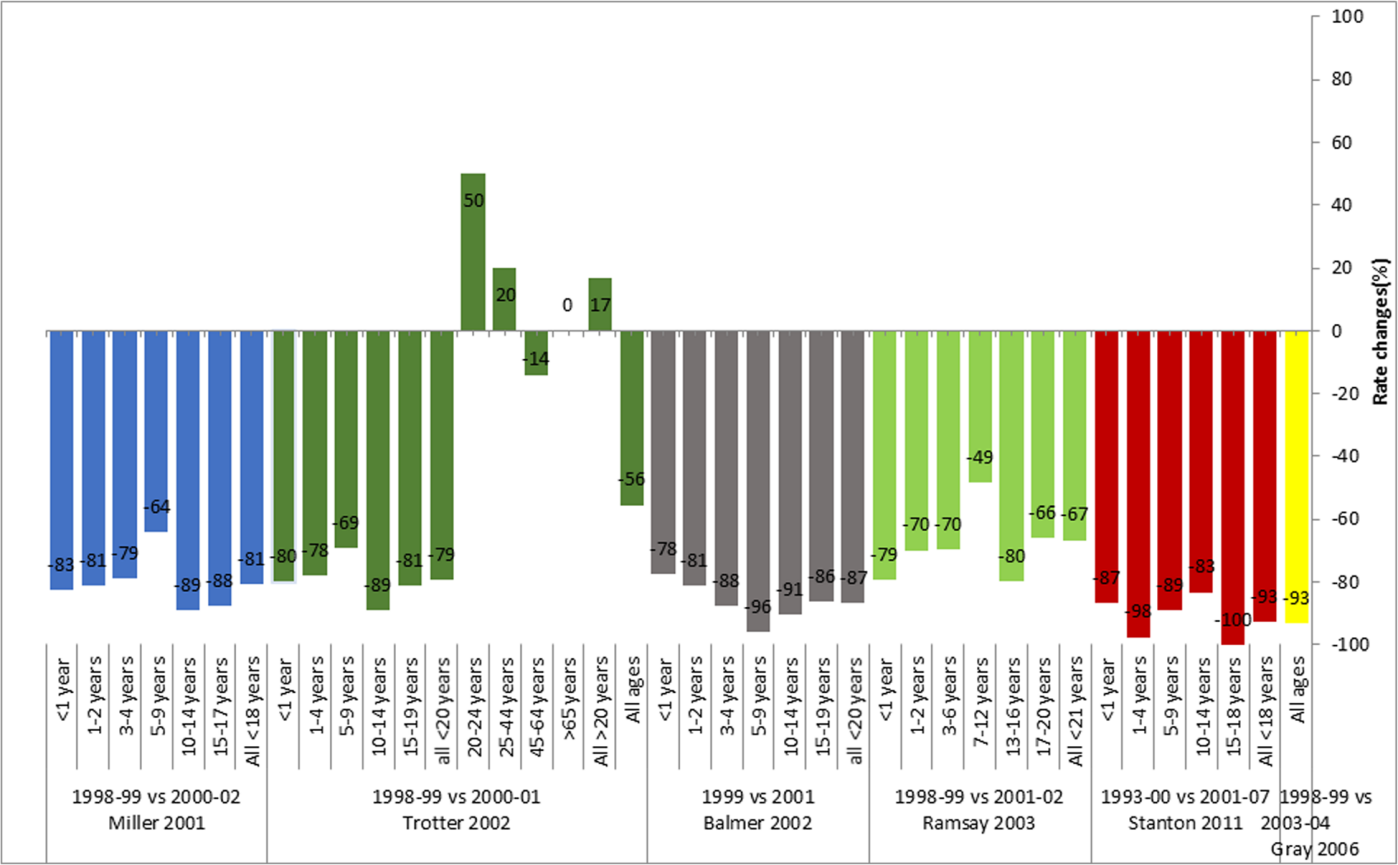
Impact of MCCV program in the UK (Incidence rate reduction comparing pre-and-post-vaccination periods). MCCV program introduced in the NIP in 1999 (at 2, 3, 4 months of age). Subsequest schedules changes were made in 2006 (at 3, 4 months), 2013 (at 2, 12 months; 13–14 years); 2015 (MCCV at 2, 12 months; MCV4 at 13–14 years); 2016 (infant MCCV doseat 2 months was removed)

The UK was the first country to introduce MCCV into their NIP in November 1999 with the schedule of 3 doses at 2, 3, 4 months of age and one catch-up dose for all children ≤18 years of age. In January 2002, the campaign was extended to include adults < 25 years of age. In 2006, the infant program was reduced to 2 doses (at age of 3 and 4 months) and a booster (MenC/Hib vaccine) added at 1 year of age. In 2013, the second infant priming dose was removed and an adolescent booster dose at age 13–14 years added to the schedule (which was then 3 months, 12 months and 13–14 years of age). In 2015, MCV4 was added to adolescent routine program replacing MCCV, and a catch-up campaign of MCV4 was offered to all aged 14–18 years and < 25 years of age attending university. In 2016, the infant dose at 3 months of age was removed from the schedule leaving the MenC/Hib combination vaccine at 12 months of age. There were 6 studies evaluating the impact of the program. Four out of six studies represented earlier post-MCCV periods before 2003 [20–23] and 2 studies covered later periods between 2006 and 2013 [24, 25]. No studies following the 2013 schedule change were available/included in the analysis. All the studies included were based on national surveillance data except one study in Merseyside, England [24]. During 1999–2001 the vaccine coverage was > 80% in all targeted age groups [20]. The largest impact was observed in children, with a reduction of MenC cases of 78–87% in infants < 1 year of age, 70–98% in children 1–4 years of age and 79–93% in all < 18/20 years of age. The largest rate reduction was observed in studies evaluating later post-MCCV periods (after 2006). The only study included in this review that evaluated impact in the initially unvaccinated population > 20 years of age during 2 years post-vaccine implementation described an increase in incidence of 17% [25] (Fig. 2).

#### Spain

**Fig. 3:**
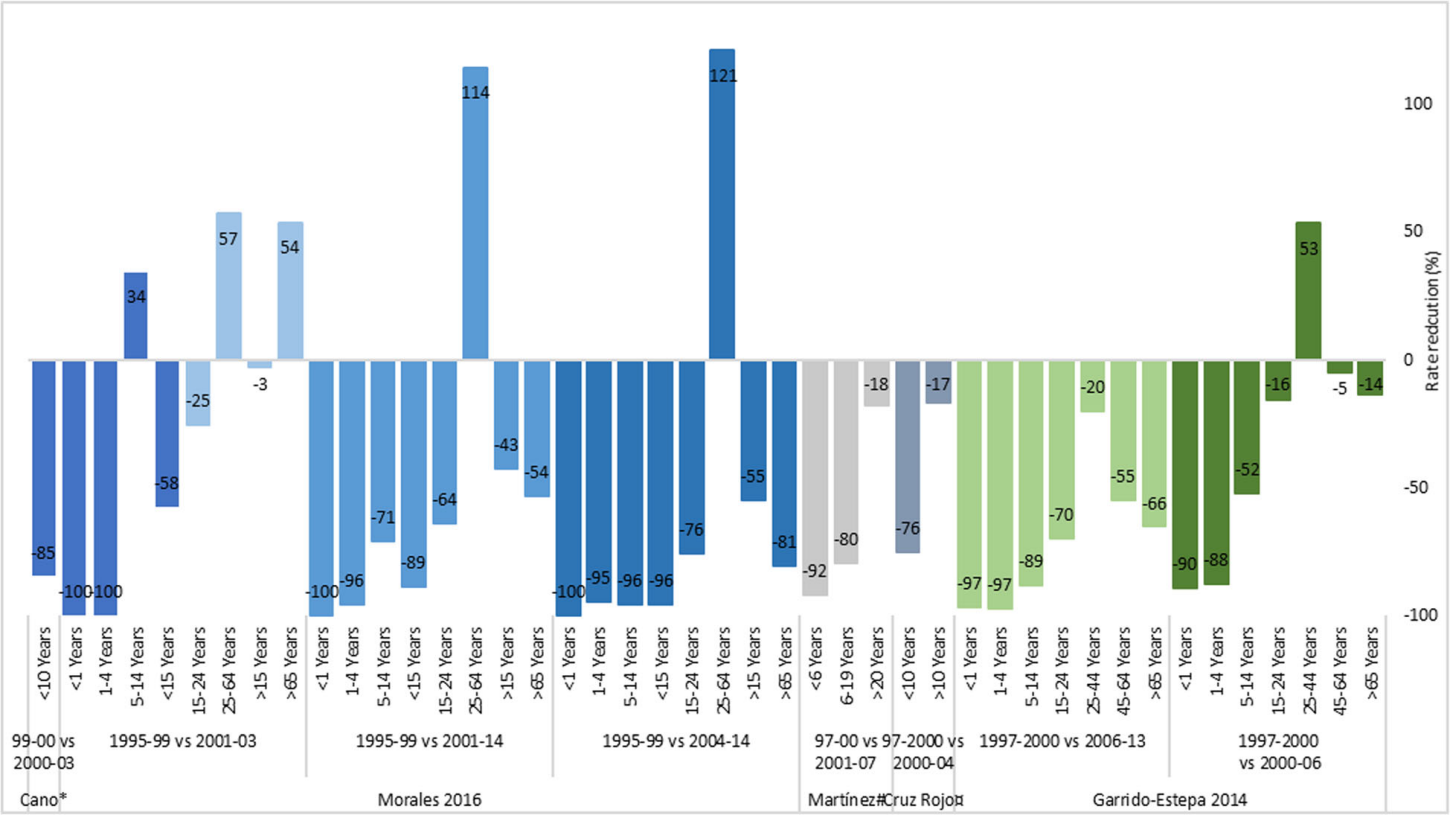
**a** Impact of MCCV program in Spain, different age groups (Incidence rate reduction comparing pre-and-post-vaccination periods). Cano*: Cano 2004; Martinez#: Martinez 2009; Rojo¤: Rojo 2005. MCCV program introduced in the NIP in 2000 (2, 4, 6 months of age). Subsequent schedule changes were made in 2006 (at 2, 4–6, 12–24 months), 2014 (at 2, 4–6, 12–24 months, 12 years). **b** Impact of MCCV program in Spain, all ages (Incidence rate reduction comparing pre-and-post-vaccination periods). MCCV program introduced in the NIP in 2000 (at 2,4, 6 months of age). Subsequent schedule changes were made in 2006 (at 2, 4–6, 12–24 months), 2014 (at 2, 4–6, 12–24 months, 12 years)

MCCV were introduced into the NIP in December 2000 with a 3 dose schedule at 2, 4 and 6 months of age along with a one dose catch-up campaign in children < 6 years old except in 3 autonomous regions where a catch-up dose was given to all < 18 years of age [60]. The catch-up campaigns differed in terms of target group, dosing schedule and periods of campaign in the different regions. In 2006, the NIP was modified to 2 doses at 2 and 4–6 months of age and a booster dose during the second year of life. One additional dose at age 12 years was added to the schedule in 2014. Since 2008 the vaccine coverage has remained > 95% for infant and > 94% for the booster dose [32]. There was a total of 7 studies from Spain (3 national studies [31–33], and one study each for Navarra [34], Galicia [35], Andalusia [36] and Catalonia regions [37]). Regardless of studies and study periods, the largest decrease of MenC cases was observed in children, with a decrease of 90–100% and 88–100% in < 1 and 1–4 years old respectively. A lesser decrease in MenC cases was generally observed with increasing age. An increase of 57–121% was observed in adults 25–64 years of age in a study in Navarra (Fig. 3a) [34]. The incidence rate was relatively low with 0.07/100,000 population (1 case) in Navarra before the MCCV program. A higher MenC incidence rate was observed in later periods (2004–2014) with 0.16/100,000 population (6 cases) compared to earlier periods (2001–2003) with 0.11/100,000 population (1 case) [34]. In summary in Spain, following routine vaccination with MCCV, MenC cases were reduced by 45–96% in all ages, with an apparent larger reduction in later periods compared to earlier post-introduction period (Fig. 3b).

#### Brazil

**Fig. 4:**
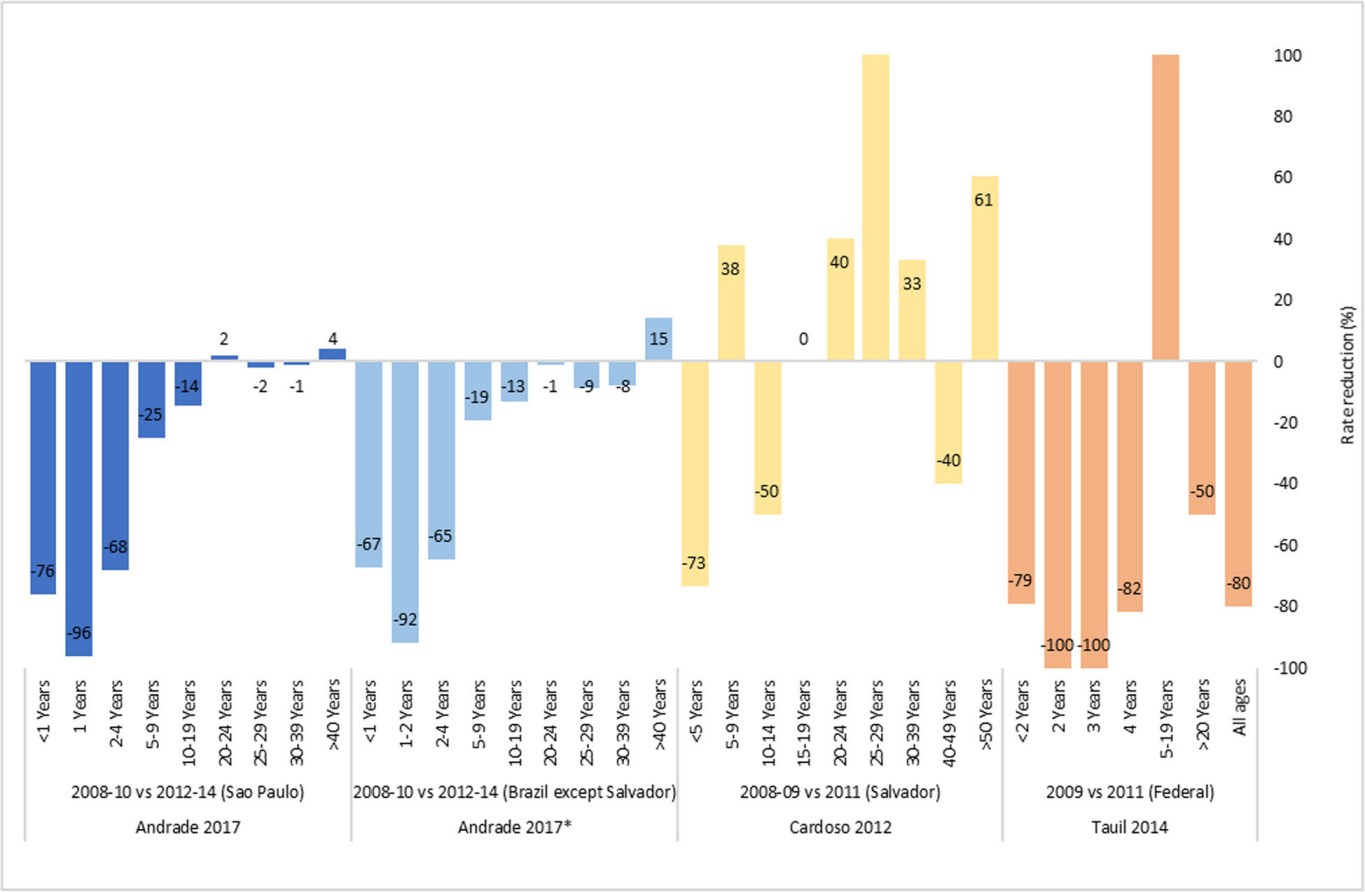
Impact of MCCV program in Brazil (Incidence rate reduction comparing pre-and-post-vaccination periods). *Brazil except Salvador. MCCV program introduced in the NIP in 2010 (at 3, 5, 12–15 months of age)

MCCV were introduced into the NIP in November 2010 with a schedule of 3 doses at 3, 5 months and 12–15 months of age. No catch-up was implemented at national level, but most states implemented catch-up vaccination for children < 2 years of age [46]. In Salvador, MCCV were introduced into the regional immunization program (Bahia federative state) in February 2010 with a 3-dose schedule at 2 and 4 months and a booster during the second year of life. One catch-up dose was given to all children < 5 years of age. Due to an unusually high number of cases and deaths, mass vaccination with a single dose of MCCV was offered to all 10–24 years old Salvador city residents during May–August 2010 [47]. Three studies were included from Brazil (Salvador City [47], Federal District [48] and the whole Brazil except Salvador city [46]). Within 4 years of MCCV introduction, there was a reduction of MenC cases of 65–100% in children < 5 years of age, with 80% reduction in all ages despite some increases in older children and young adults (Fig. 4).

#### Other countries

##### Canada

**Fig. 5:**
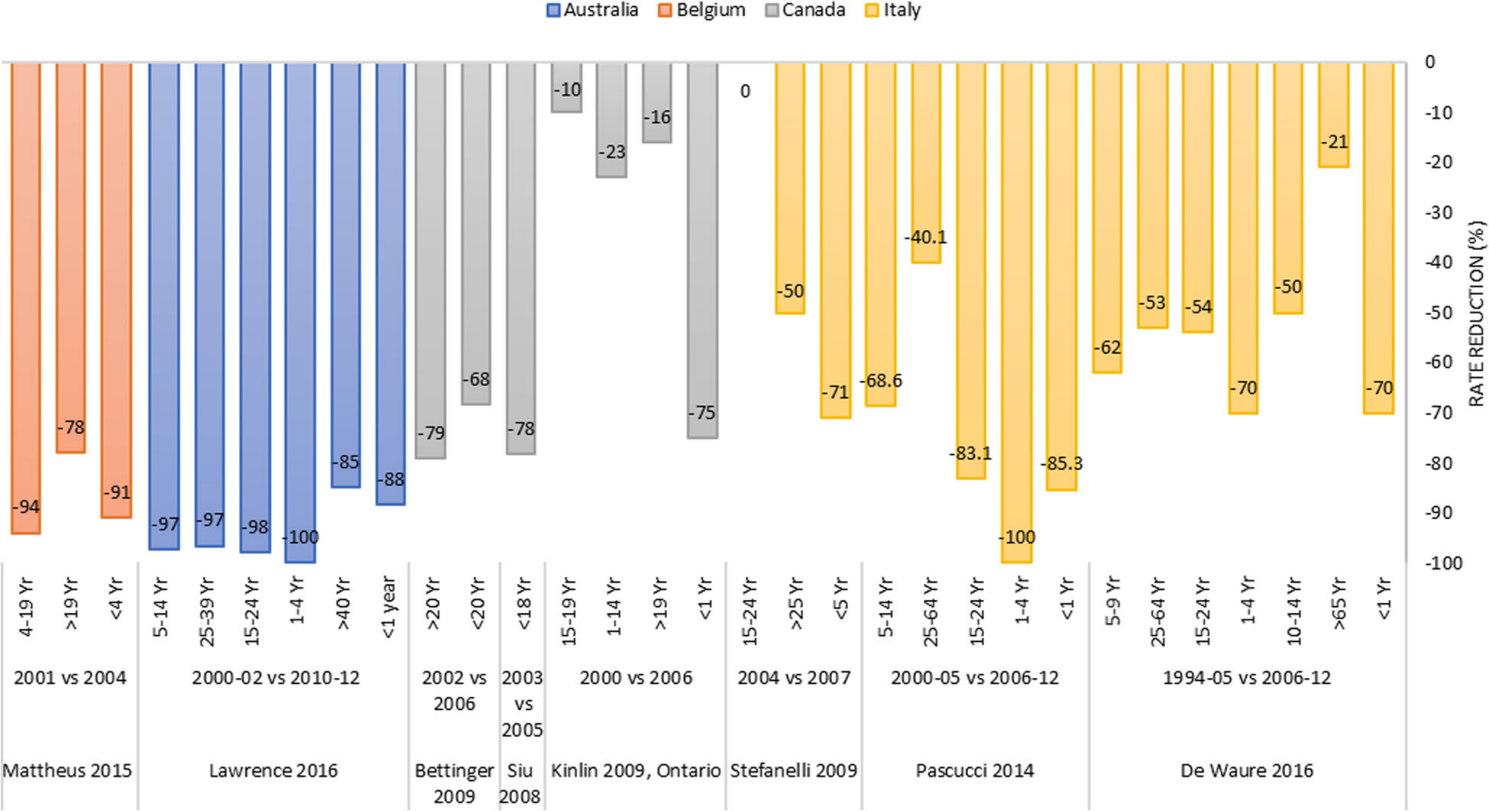
Impact of MCCV program in other countries (Incidence rate reduction comparing pre-and-post-vaccination periods). Australia: MCCVintroduced in 2003 (at 12 months of age); Belgium: MCCV introduced in 2002 (at 12–15 months of age); Canada: MCCV introduced in 2001–2005 (at 2, 4, 6 months of age); Italy MCCV program introduced in the NIP in 2000 (2, 4, 6 months of age)

Differences in local disease epidemiology and programmatic considerations led to a variety of schedules being used in different provinces. MCCV were introduced into the immunization programs of all provinces and territories except Nunavut during 2001–2005 with 3 doses at 2, 4, and 6 months of age, and a modified schedule (2 doses at least 4 weeks apart for children 4–11 months old) [44]. However, during 1999–2001, mass vaccination campaigns using non-conjugate or conjugate polysaccharide vaccines were implemented to contain regional or local outbreaks [61]. All estimates from the 4 studies included described a reduction of MenC cases regardless of region, age and study period. Three studies described a reduction of 10–79% in early post-MCCV periods (before 2006) (Fig. 5) [41–43]. However, one study describing later periods, between 2002 and 2012 depending on the provinces, reported 79–94% reduction in all ages. The incidence decreased from 0.10 to < 0.05 /100,000 population in all ages in 2012, 8 years following the start of the program with a significant reduction of 14% per year. The largest decrease (83%) was observed in the 15–24 years age group (data not shown in the Fig. 5) [44]. All provinces and territories in Canada have since introduced a routine adolescent meningococcal vaccine dose (either MCCV or MCV4) in 2007, with the exception of Alberta (introduced in 2010) and Quebec (2013) [44].

##### Australia

MCCV were introduced into the NIP in 2003 with a single dose at 12 months of age and a catch-up dose for individuals aged 2–19 years. Seventy-six percent of the eligible population received the vaccine between 2003 and 2012. The highest coverage, 93%, was in children < 1 year of age and the lowest, 22%, in school leavers (18–19 years of age). Two studies included reported a significant reduction of 85–100% in different age groups (Fig. 5). This decrease in incidence was observed just 1–2 years after the program start and the low incidence rate was sustained in the following years (data not shown) [54, 55].

#### Europe

##### Belgium

MCCV were introduced into the Belgian NIP in 2002 as one-dose at 12–15 months of age. A one-dose catch-up campaign was implemented for all children 1–5 years old in the Wallonia region and for 1–18 years old in the Flanders region because there had been a steeper rise in incidence in Flanders [59]. We identified a single study describing the impact reporting a significant reduction in cases within all age groups from 2001 to 2004 with a 92% reduction in Flanders and 77% reduction in Wallonia (Fig. 5) [59].

##### Germany

MCCV were introduced into the routine NIP in 2006 as a single dose in the second year of life. While a catch-up dose was recommended in all children < 18 years of age, a coordinated catch-up campaign was not undertaken. Vaccine coverage was 70% in children entering school in 2010. The annual incidence rate decreased from 0.26 to 0.10/100,000 in < 25 years of age between 2003 and 2010. Thereduction was more pronounced in regions with higher vaccine coverage compared to those with low vaccine coverage and in vaccine eligible ages with the annual reduction rate of 19% in 1–5 years old compared to 9% in individuals 15–24 years olds [58].

##### Italy

MCCV were introduced into regional immunization programs during 2005–2007 with the schedule of a single dose at 13–15 months of age and a catch-up at 11–18 years of age [50]. The vaccine coverage varied widely from 37 to 93% among regions. A significant reduction of MenC cases in all ages was observed across all 3 out of 4 studies included with the highest impact in children < 5 years of age (70–100% reduction) and the least decrease in adults over 65 years old (21%) during 2006–2012 compared to the period before 2005 (Fig. 5) [50, 51, 53]. One study reported the decrease in all ages but with a higher impact in children < 5 years old between 2008 and 2009 and 2012–2013 [52].

##### The Netherlands

MCCV were introduced into the NIP as a single dose at 14 months of age following a mass MCCV vaccination campaign in 2002 for all 1–18 years old. Vaccine coverage reached 94% in the target population. There was a significant decrease of 99% in the vaccine-eligible population and 93% in non-vaccine-eligible populations from 1998 to 2002 to 2008–2012 [57]. The overall incidence rate decreased significantly from 17.3/100,000 population during the epidemic in 2001 to 0.06/100,000 population in 2012 [56] (Data not shown).

### MCCV Effectiveness

**Fig. 6:**
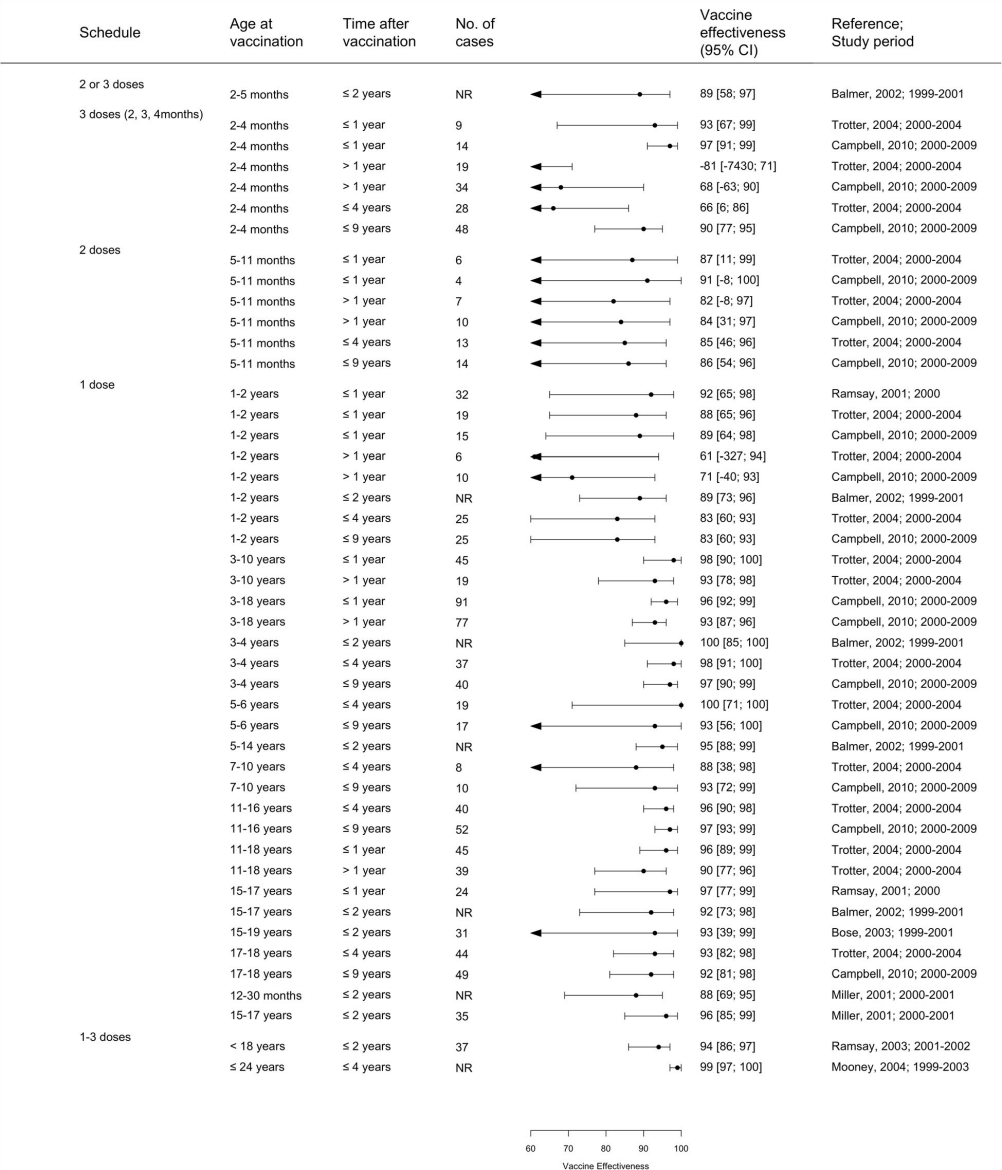
Vaccine Effectiveness estimates of MCCV by doses received and by age at vaccination in the UK. Footnotes: 1–3 doses (3 doses at 2, 3, 4 months); 2 doses for 5–11 months old; 1 dose for ≥ year. Abbreviation: NR: Not reported

**Fig. 7:**
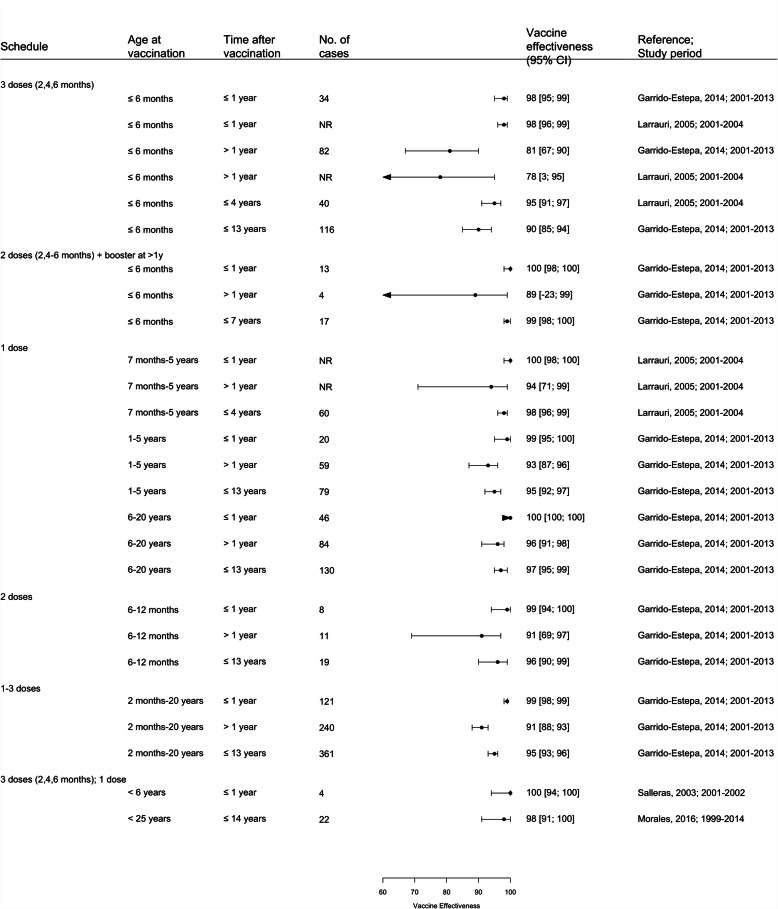
Vaccine Effectiveness estimates by doses received and by age at vaccination of MCCV in Spain

A total of 17 studies from 5 countries were included in the vaccine effectiveness review, 10 used the screening method to evaluate VE [20, 21, 26–30, 32, 34, 38, 39, 47, 53], 5 were retrospective cohort studies (Spain and Quebec-Canada) [22, 29, 33, 34, 45] and 2 were case-control studies [26, 49] (Supplementary Table 1). Most of the studies using cohort or case-control designs, also estimated VE using the screening method. Most studies described several VE estimates according to different age groups, schedules and time since vaccination. A total of 147 VE estimates were available (53 from the UK, 79 from Spain, 12 from Canada, 2 from Brazil and 1 from Italy) (Table 1, Fig. 6, Fig. 7, Supplementary Figure 1). Of 147 VE estimates, 138 are statistically significant. A total of 146 of the 147 estimates described positive vaccine effectiveness ranging from 34% (95% CI: − 168; 83) (in children < 1 year for > 2 years post-vaccination, Canada) to 100% (95% CI: 100; 100) (5 estimates from UK, Spain and Brazil) [20, 30, 32, 33, 47] and only one estimate reported negative effectiveness (− 81%; 95% CI: − 7430; 71) in UK children more than 1 year following vaccination at 2-3-4 months of age [30]. The same study reported a VE of 93% (95% CI: 67%;99%) for these children within the 12 months immediately following vaccination [30]. Campbell and colleagues [27] reported data from an extension of this earlier study incorporating a larger number of subjects vaccinated at 2-3-4 months of age with a VE of 68% (95% CI: − 63; 90) more than 1 year following vaccination. Of 146 positive VE estimates, 4 from the UK, 1 from Spain and 3 from Canada showed statistical non-significance.

**Table 1:** MCCV effectiveness within and over 1 year following vaccination of included studies

Country, study period (References)	Schedule and at age at vaccination	Vaccine effectiveness (95% CI); time post-vaccination	
		≤1 year	> 1 year
**3 doses**			
UK, 2000–2004 (Trotter 2004) [30]	Routine 3 doses (at 2, 3 and 4 months)	93% (67 to 99)	−81% (−7430 to71)
UK, 2000–2009 (Campbell 2010) [27]	Routine 3 doses (at 2, 3, and 4 months)	97% (91 to 99)	68% (− 63 to 90)
Spain 2001–2013 (Garrido-Estepa 2014) [32]	Routine 3 doses (at 2,4 and 6 months)	98% (95 to 99)	81% (67 to 90)
Spain 2001–2013 (Garrido-Estepa 2014) [32]	Routine 3 doses (at 2,5 and > 12 months)	100% (98 to 100)	89% (− 23 to 99)
Spain 2001–2013 (Garrido-Estepa 2014) [32]	Routine 1–3 doses (at < 20 years)	99% (98 to 99)	91% (88 to 93)
**2 doses**			
UK, 2000–2004 (Trotter 2004) [30]	Catch-up 2 doses (2 doses at 5-12 months)	87% (11 to 99)	82% (−8 to 97)
UK, 2000–2009 (Campbell 2010) [27]	Catch-up 2 doses (2 doses at 5-12 months)	91% (− 8 to 100)	84% (31 to 97)
Spain 2001–2013 (Garrido-Estepa 2014) [32]	Catch-up 2 doses (at 1–5 years)	99% (94 to 100)	91% (69 to 97)
**1 dose**			
UK, 2000–2004 (Trotter 2004) [30]	Catch-up 1 dose (at 1–2 years)	88% (65 to 96)	61% (− 327 to 94)
UK, 2000–2004 (Trotter 2004) [30]	Catch-up 1 dose (at 3–4 years)	98% (90 to 100)	93% (78 to 98)
UK, 2000–2004 (Trotter 2004) [30]	Catch-up 1 dose (at 11–16 years)	96% (89 to 99)	90% (77 to 96)
UK, 2000–2009 (Campbell 2010) [27]	Catch-up 1 dose (at 1–2 years)	89% (64 to 98)	71% (− 40 to 93)
UK, 2000–2009 (Campbell 2010) [27]	Catch-up 1 dose (at 3–18 years)	96% (92 to 99)	93% (87 to 96)
Spain 1999–2004 (Larrauri 2005) [39]	Catch-up 1 dose (at 7 months-5 years)	100% (98 to 100)	94% (71 to 99)
Spain 2001–2013 (Garrido-Estepa 2014) [32]	Catch-up 1 dose (at 6–12 months)	99% (95 to 100)	93% (87 to 96)
Spain 2001–2013 (Garrido-Estepa 2014) [32]	Catch-up 1 dose (at 6–20 years)	100% (100 to 100)	96% (91 to 98)

Four studies (2 from each of the UK and Spain) specifically evaluated the VE over time (≤1 year versus > 1 year) to determine if waning protection occurred (Table 1) [27, 30, 32, 39]. While there were, to some extent, overlapping 95%CI, the VE was generally higher when evaluated within 1 year of vaccination (range of 78 to 100%) compared to those for ≥1 year since vaccination (range of − 81 to 98%) regardless of age at vaccination, dosing schedule or program (routine or catch-up programs). Wide confidence intervals were usually observed in analyses evaluating VE > 1 year post-vaccination (due to small number of cases) (Figs. 2, 3, supplementary figure 1). One study in Spain reported the waning VE of 50% after 10 years in young infants while those vaccinated with one dose at 12–19 years old maintained the effectiveness [38]. One study in Canada also evaluated VE over time, reporting a decline from 83 to 34% when measured within 2 years and > 2 years after vaccination, respectively. The VE of catch-up doses in adolescents ranged from 92 to 98% [45]. No significant differences in VE estimates were observed between countries, vaccine schedules and population studies, but routine schedules where at least one dose was administered during the second year of life or catch up programs had better sustained VE in the long term. Meta-analysis was not performed due to heterogeneity in study characteristics, vaccine schedules evaluated and the study populations.

## Discussion

This is the first literature review confirming the overall VE and impact of MCCV when introduced into country wide NIPs. MCCV were originally licensed on the basis of the serological correlate of protection, since the low incidence rate of MenC IMD precluded the conduct of efficacy trials. Although MCCV are proven highly immunogenic and elicit Serum Bactericidal Antibody (SBA), in the absence of clinical efficacy studies, post-licensure effectiveness studies are of particular relevance [62, 63].

This review has illustrated high and statistically significant VE of MCCV in 138 out of 147 estimates. The overall VE ranged from 38 to 100% in vaccine eligible populations regardless of the time since vaccination, or the dosing schedule (routine or catch-up doses) except for one estimate of − 81% (95% CI: − 7430 to 71) at > 1 year following a 3-dose infant schedule. The majority of VE estimates were from children < 5 years of age due to NIPs often being implemented in infants and toddlers. These VE estimates tended to be higher than in adolescents, particularly during the first year following vaccination and may be related to the higher uptake often seen in the younger age groups. It is not unexpected because persistence of immune responses is greater in adolescents compared to infants during the clinical studies. Regarding VE over time, the estimates were consistently higher for those evaluated within 1 year of vaccination, relative to those evaluated after 1 year, regardless of the study type, dose and country [38]. These findings are consistent with decreases in the proportions of individuals with protective SBA titres overtime following vaccination in clinical studies [64–66]. The requirement for the maintenance of protective levels of circulatory anti-MenC SBA to confer continued protection was clearly demonstrated following MCCV implementation by the decreasing VE, especially where infant vaccination alone was employed. This was subsequently understood to be due the insufficiency of immunological memory to provide a response of sufficient rapidity to prevent infection and mirrored that already observed following Hib vaccine implementation [67, 68].

From a public health perspective, significant and immediate reduction of MenC incidence was consistently observed in vaccine eligible ages in all countries with high vaccine uptake either routine only and / or with catch-up campaign. The reduction in non-vaccine eligible ages (especially population > 65 years) through herd protection was generally observed following 3–4 years of introduction.

Along with changes in meningococcal disease epidemiology, vaccination policies have changed over time, either as changes in schedule or more recently due to switching from monovalent conjugate to quadrivalent conjugate vaccines. In the UK, Ireland and Spain, a 3 dose infant series without a toddler booster dose (3 + 0) was initially introduced coupled with a mass catch up campaign. Significant reductions of MenC cases first in vaccine eligible followed by non-vaccine eligible ages were observed. The vaccine schedule was changed to a 2 dose primary series and a toddler booster dose (2 + 1) in the UK and Spain in 2006 [69] and in Ireland in 2008. Despite the changes, continuous decreases of MenC cases were observed. In England, the reported cases of MenC IMD had significantly reduced from 883 cases in 1998–1999 to about 30 cases in 2014–2015 (~ 97% reduction) with 0–1 cases in infants during 2012–2016. In Ireland, reported MenC cases decreased from 139 cases in 2000 to 0–1 cases in 2012–2013 (~ 100% reduction). In Spain, about 90–100% reduction of MenC cases were observed in children < 5 years old [31, 32, 34, 37]. However, following recent schedule changes from 2 + 1 to single doses in infants, toddlers and adolescents (1 + 1 + 1) in the UK in 2013 and in Ireland in 2015 and the subsequent removal of the infant dose in the UK in 2015, re-emergence of MenC has been observed in the UK and Ireland. In the UK, the total reported MenC cases increased from 0 to 33 cases in infants and from 33 to 64 cases in all ages between 2012 and 2013 and 2017–2018 [70]. The driver of this increase is not fully clear but is likely associated with the increased reliance on herd protection as a driver of population control rather than individual or direct protection. Other countries such as Australia, Belgium, Italy, the Netherlands, Canada, Germany and France have introduced a single dose of MCCV in infants or toddlers into the NIP along with mass catch-up campaigns. Countries reaching a high vaccine uptake soon after introduction resulted in a corresponding quick and significant reduction in MenC cases. This is exemplified by Australia, where only 3 cases were reported in 2016 following the 2003 introduction of a single dose of MCCV at 12 months of age with a catch-up programme for 2–19 years old [71]. Similarly in the Netherlands, zero and 6 cases were reported in children < 14 years and all ages respectively in 2016 [72]. In France, despite the introduction of MCCV since 2010, the vaccine uptake was still only 39% in children 5 months of age and at 73% in children < 2 years of age in January 2017. Meanwhile a significant increase of MenC was observed especially in infants between 2010 and 2016. As consequences, routine infant schedule was changed from one dose (<12 months of age) to 2 doses (5 and 12 months of age) in May 2017. An immediate decrease in MenC cases was observed in infants while cases continued to increase and became the most prevalent serogroup in adults > 25 years of age [73]. This resulted in the implementation of a catch-up programme for 1–24 years old unvaccinated individuals in 2018 [74]. In Germany, a gradual decrease in MenC cases was already observed prior to the introduction of the program. However, the decrease was more pronounced and accelerated in regions with high vaccine uptake (> 74%) compared to those with lower uptake (< 61%) [58]. In Italy, a significant reduction was observed immediately following MCCV introduction whilst the vaccine uptake varied from 35 to 75% according to regions. Recently there were some clusters of MenC cases described in adolescents in 2015–2016 in Tuscany where the vaccine uptake was < 70% in 2014 [75].

In recent years, meningococcal serogroup W (MenW) has been an increasing cause of disease across all age groups and in many geographically dispersed countries [69, 76, 77]. As a direct consequence quadrivalent ACWY vaccines have been added as a single adolescent dose to induce herd protection into the immunization programs of countries including the UK (2015), Ireland (2016), the Netherlands (2018), Spain and Australia (2019). It is also noteworthy that a quadrivalent meningococcal ACWY vaccine, without prior monovalent vaccine use, was introduced in the NIP in the United States in 2005 with one dose at the age of 11–12 years adolescent [78]. In 2010, a booster dose was added at age of 16 years following a study describing the waning effectiveness beyond 5 years post-vaccination [79].

This review had several limitations. First, a meaningful meta-analysis on VE estimates was not feasible. This was because the studies were heterogenous in terms of schedules, age at vaccination and study methods used [80]. In addition, immunization programs (choice of vaccine and scheduling) were constantly changing over time even within a country along with serogroup evolution and affected populations. Secondly, the review included only published papers. In most European countries, the latest disease data were reported only in publicly available reports from national and supranational surveillance but not necessarily in published papers. Therefore, the data from Ireland, France were not captured in the analysis while they are detailed and described in the discussions. In addition, the majority of data included in this review for UK and Germany are now historical and relate to the period before 2010 (Supplementary Table 1) with limited contemporary data reflecting the currently employed schedules which have changed from those originally implemented. Thirdly, 13 out of 17 VE studies included in the review used exclusively the screening method which compared the national vaccine coverage for MCCV to MenC cases in vaccine eligible ages in the general population where individual vaccination status is known. This method is usually non-expensive and practical since it allows rapid estimation of VE using readily available surveillance data. However, it is relatively sensitive to errors (even minor) in input parameters such as incidence of the disease and the accuracy of the vaccine coverage rate in the population and relies on the availability of high quality surveillance data for these inputs. Usually the incidence data are collected for purposes other than for the study specifically and the age-specific vaccine coverage estimates may not be sufficiently accurate in many study settings [68, 81]. While MenC cases are severe enough to have an appropriate diagnosis for all suspected cases and appropriately reported, the vaccine coverage rate in vaccine eligible ages could certainly be a limitation for several studies. Lastly, three MCCV have been licensed and the results have been collectively presented irrespective of the vaccine used. Whilst in some countries, the results discussed here have been generated by a single MCCV, there are other countries where multiple vaccines have been used concurrently or consecutively during a particular study period and even countries where MCCV used has varied on a regional basis. Differences in the immunogenicity of MCCV vaccines have been reported in terms of the proportions achieving protective SBA levels and the persistence of these protective levels which could translate to differences in VE [82, 83].

## Conclusions

MCCV are highly effective, showing a substantial and sustained decrease in MenC cases in countries using them and achieving a high vaccine uptake. VE waned over time, but protection was maintained at the population level in countries with high vaccination coverage rate especially where catch-up campaigns were originally and effectively implemented. Epidemiology of meningococcal disease is in constant transition, and vaccination programs are extending towards adolescent programs to impart herd protection and higher valent vaccines due to the recent increases in serogroups not included in MCCV such as MenW and Y. Continuous monitoring of meningococcal disease and their genomic profile is an essential part in understanding the disease evolution along with the vaccination programs.

## Supplementary Information

**Additional file 1.** Full protocol.

**Additional file 2: Supplementary figure 1.** Vaccine effectiveness of MCCV in other countries. **Supplementary Table 1.** Summary characteristics of the studies included in the review.

## Data Availability

Not applicable.

## Abbreviations

DT: Diphtheria toxoid
HR: Hazard ratio
IMD: Invasive meningococcal disease
IRR: Incidence rate ratio
MCCV: Monovalent MenC conjugate vaccines
MCV4: ACWY meningococcal conjugate vaccines
MenC: Meningococcal serogroup C
NIP: National Immunization Program
OR: Odd ratio
RR: Relative risk
TT: Tetanus toxoid
VE: Vaccine Effectiveness
