# Supplementary material for: Systematic literature review of the impact and effectiveness of monovalent meningococcal C conjugated vaccines when used in routine immunization programs
Source: BMC Public Health. 2020 Dec 9;20:1890. doi: 10.1186/s12889-020-09946-1 (PMC7724720; doi:10.1186/s12889-020-09946-1)
Supplement: Supplementary file 1 — Additional file 1. Full protocol. [file 12889_2020_9946_MOESM1_ESM.pdf]

Systematic literature review and analysis: Worldwide impact and effectiveness of Meningococcal C vaccination when used as part of routine immunization programs

Protocol

Version	Date	Previous version	Changes made since previous number
1	03/11/2017	NA	NA
2	10/11/2017	1	Protocol updated after communication with Pfizer

Table of Contents

Table of Contents **2**

1. Background **4**

2. Objectives **4**

3. Methods **4**

3.1. Eligibility criteria **4**

3.1.1. Inclusion criteria 4

3.1.2. Exclusion criteria 5

3.2. Information sources **5**

3.3. Data selection **6**

3.3.1. PRISMA guidelines 6

3.3.2. Data management and storage 6

3.3.3. Selection of studies 6

3.3.4. Reference checking and hand searching 7

3.3.5. Double up of study reports 7

3.4. Data extraction, analysis and tabulation **7**

3.5. Results **9**

3.6. Quality assessment **9**

4. Analyses and reporting **10**

5. References **11**

Acronyms

Hib	Haemophilus influenzae type b
HR	Hazard ratio
IMD-C	invasive Meningococcal Disease, serogroup C
IRD	Incidence rate difference
IRR	Incidence rate ratio
MCCV	Meningococcal C Conjugate Vaccination
MenACWY	Meningococcal group A, C, W-135, and Y Conjugate Vaccine
NIP	National Immunization Program
OR	Odds ratio
PRISMA	Preferred Reporting Items for Systematic reviews and Meta-analysis
RR	Relative risk
SLR	Systematic Literature Review
VE	Vaccine effectiveness

1. Background

Meningococcal disease results from the infection caused by gram-negative bacterium *Neisseria meningitidis*, frequently referred to as meningococcus. While best known as a cause of meningitis (inflammation of the meninges), infection can lead to a wide range of manifestations including sepsis and pneumonia. Meningitis and meningococcemia (bloodstream infection), are severe causes of morbidity, mortality, and disability in both developed and under-developed countries and can cause a serious burden on the public health system (1).

Six subtypes (namely serogroups A, B, C, W-135, X, and Y) of *N. meningitidis* are responsible for most cases of meningococcal disease worldwide (1). Meningococcal vaccines, available for several subtypes, have reduced disease incidence in many countries. Meningococcal C conjugate vaccination (MCCV) has been very effective for the control of Meningitis C (2), but meningococcal disease has a dynamic epidemiological profile and the re-emergence of the W-serogroup has driven reconsideration of existing vaccination recommendations towards the quadrivalent meningococcal group A, C, W-135, and Y conjugate vaccine (MenACWY) (3).

2. Objectives

The objective of this project is to perform a systematic literature review (SLR) to describe:

- the impact and effectiveness of MCCV on hospitalizations of children, adolescents, or adults worldwide for invasive meningococcal disease (IMD-C), in settings where MCCV has been integrated in routine vaccination programs;
- the epidemiological trends of IMD-C hospitalizations, in settings where MCCV has been integrated in routine vaccination programs.

3. Methods

3.1. Eligibility criteria

3.1.1. Inclusion criteria

The following criteria for inclusion will be used to select studies to be included in the review:

i. Population: all ages
ii. Geography: countries where MCCV has been introduced in routine vaccination programs, i.e. included in the National Immunization Program (NIP).
iii. Intervention: MCCV

iv. Outcome of interest: hospitalization for IMD-C. Primary care might be included after reviewing the available evidence for hospitalizations.
v. Summary measures of interest:
   - For effectiveness: vaccine effectiveness (VE), relative risk (RR), odds ratio (OR), hazard ratio (HR)
   - For impact: incidence rate ratio (IRR), incidence rate difference (IRD)
vi. Setting: single or multiple hospital
vii. Study designs: observational studies (e.g. cohort, case-control, surveillance-based, ecological)
viii. Time period: January 2001 – October 2017
ix. Languages: English, French, Spanish, Portuguese, Dutch, German, Italian.

3.1.2. Exclusion criteria

Studies with any of the following criteria will be excluded from the review:

i. Data from countries where MCCV is not part of the routine vaccination program.
ii. Health-economic impact studies.
iii. Models/simulations/extrapolations.
iv. Awareness and/or acceptability studies
v. Studies reporting on combination vaccines (e.g. MenACWY) (exception: studies reporting on the Hib/MenC combination vaccine in the UK will be included)
vi. Long-term follow-up studies of clinical trials
vii. Review papers
viii. Immunogenicity studies
ix. Studies reporting on the use of the vaccine for the control of disease outbreaks

3.2. Information sources

We will conduct a literature search in the databases of MEDLINE (via PubMed), LILACS, and SCIELO to obtain peer-reviewed, scientific publications meeting the objectives and inclusion criteria of interest to the review.

The search string will consist of vaccine terms, outcome terms and effect measurement terms. For example, for the search in PubMed the following terms and strings will be used:

<u>#1 vaccine terms</u>

((Meningococcal OR Meningit*) AND Conjugate AND (“Serogroup C” OR MCC) AND (vaccine OR vaccination))

#2 outcome terms

(Hospital OR Hospitalization OR Hospitalisation OR admission OR invasive meningococcal disease OR IMD)

#3 effects measurements terms

(“Vaccine effectiveness" OR “odds ratio” OR OR OR “Relative risk” OR RR OR “Hazard ratio” OR HR OR Incidence OR rate OR trend OR epidemiology OR evolution)

Full search

#1 AND #2 AND #3

In PubMed, the search will be filtered to include human studies only.

Grey literature will also be searched using targeted expressions in a generic search engine (e.g. Google), in addition to targeted searches in public health institutions websites (e.g. US-CDC, ECDC, WHO and WHO regional offices, and national public health agencies of relevant countries).

3.3. Data selection

3.3.1. PRISMA guidelines

The Preferred Reporting Items for Systematic reviews and Meta-analysis (PRISMA) guidelines will be applied to optimally select, analyse and report on studies in this systematic review (4). A flowchart/selection tree will be created to record and illustrate the flow of information through the different stages of the review.

3.3.2. Data management and storage

The results of the literature search will be imported into Endnote© and Rayyan©.

3.3.3. Selection of studies

The selection process will take place in two steps.

Screening:

In the first step, two reviewers will screen the titles and abstracts of records identified through database searches for their relevance, based on the inclusion/exclusion criteria. In case of doubt, a third opinion will be sought from another reviewer. Publications for which it is not clear from the title or abstract whether they meet the screening exclusion/inclusion criteria will be included for full-text reading.

Similarly, an internet search will be performed to find and retrieve public health and/or surveillance reports with data on any of the outcomes and their relevance for inclusion will be determined by a single reviewer.

Eligibility:

The second selection step will be a full-text review of the articles retrieved in the first screening step by a single reviewer. In this second step, a track record of the selection process will be maintained with the reasons for exclusion.

A list of the review publications references will be prepared and sent to Pfizer for review before P95 proceeds to data extraction.

3.3.4. Reference checking and hand searching

We will hand search the reference lists of reviews, guidelines, meta-analyses and eligible studies retrieved from the second selection step to identify potential additional studies.

3.3.5. Double up of study reports

Where more than one report on the same study (population) is identified (e.g. temporal reporting), data collected from these reports will be merged into a single entry.

3.4. Data extraction, analysis and tabulation

Data from the eligible full-text papers identified in the second selection step will be extracted using a standardized extraction form to ensure that all relevant data are collected systematically. The section of the pdf manuscript from where data will be collected, will be noted and/or highlighted. Data will be stored in MS Excel, with separate tabulations for impact and effectiveness of MCCV on hospitalizations. The template will be piloted with 10 studies and modifications made if necessary.

Data extraction will be carried out by a single reviewer; re-extraction of 10% of the papers will be done by a second reviewer.

The following information will be extracted:

- Reference (PMID)

- Study aim:
    - Impact
    - Effectiveness
    - Descriptive
- Methods/design
    - Surveillance
    - Screening method
    - Case-control
    - Cohort
    - Ecological
- Prospective or retrospective
- Case definition
- Diagnostic method
- Country
- Country coverage
    - Nationwide
    - Regional
- Setting
    - Single Hospital
    - Multi Hospital
    - Information on hospital(s)
- Population
    - Age group(s)
    - Population size
        - Number of individuals
        - Number exposed/unexposed, cases/controls (as appropriate for study design)
        - Number of cases
- Study period
- Outcomes
    - MCCV hospitalization

- Summary measures
    - Effectiveness: VE, OR, RR, HR
    - Impact: incidence before/after introduction, IRR, IRD

3.5. Results

Tables and results will be generated separately for impact and effectiveness of MCC vaccination, and will be stratified and tabulated by age group and country. Proposed dummy tables are shown below (Table 1 and 2).

The framework by Halloran et al. 2009 (5) will be used to define effectiveness and impact. Effectiveness has been split into direct effects (measured by comparing vaccinated and unvaccinated persons belonging to the same population and exposed to the same vaccination program) and indirect effects (population-level effects of widespread vaccination, as a result of reduced transmission; i.e. herd immunity). Impact expresses the overall effect of the vaccination program on an entire population, including vaccinated and unvaccinated individuals (5;6).

Example of effect measures for effectiveness are VE, RR, OR, and HR. Examples of effect measures for impact are IRR and incidence rate differences.

Data will be extracted as reported in the publication and we will attempt to reclassify as per Halloran et al. 2009 (5) and Hanquet et al. 2013 (6).

Table 1. Effectiveness of MCCV on IMD-C hospitalizations

Reference	Country	Region(s)	Study period	Study design	Data source	Age group	Year of introduction	Case definition	Diagnostic method	Effect measure
										VE/RR/ OR/HR

Table 2. Impact of MCCV on IMD-C hospitalizations

Reference	Country	Region(s)	Study period	Study design	Data source	Age group	Year of introduction	Incidence before/after	Case definition	Diagnostic method	Effect measure
											VE/RR/ OR/HR

3.6. Quality assessment

No formal quality assessment of the studies or evidence grading will be done.

4. Analyses and reporting

The results of the systematic review will be reported as a narrative review in the form of a scientific publication, and including where feasible a quantitative assessment.

The outline of the scientific publication will follow the PRISMA guidelines (4):

1. Introduction
    a. Rationale
    b. Objectives
2. Methods
    a. Protocol
    b. Eligibility criteria
    c. Information sources
    d. Search
    e. Study selection
    f. Data collection process
    g. Data items
3. Results
    a. Study selection (Selection Tree)
    b. Effectiveness of MCCV on hospitalizations for IMD-C
    c. Impact of MCCV on hospitalizations for IMD-C
    d. Epidemiological trends of IMD-C hospitalizations
4. Discussion
    a. Summary of findings
    b. Limitations
    c. Conclusions

The studies characteristics and summary measures will be summarized in standard tables. The decision to perform a subsequent meta-analysis will be made based on the available results, after data extraction, and planned accordingly in a separate document that will be annexed to this protocol.

5. References

1. Harrison L.H. (2010). Epidemiological profile of meningococcal disease in the United States. *Cilin Infect Dis.* 50 (Suppl 2): S37-S44.
2. Prasad K., Karlupia N. (2007). Prevention of bacterial meningitis: an overview of Cochrane systematic reviews. *Respir Med.* 101: 2037-2043.
3. Zahlanie Y.C., Hammadi M.M., Ghanem S.T., Dbaibo G.S. (2014). Review of meningococcal vaccines with updates on immunization in adults. *Hum Vaccin Immunother.* 10: 995-1007
4. Moher D., Liberati A., Tetzlaff J., et al. (2009). Preferred reporting items for systematic reviews and meta-analyses: the PRISMA statement. *Plos Med.* 6: e1000097.
5. Halloran E., Longini I.M., Struchiner C.J. (2009). *Design and analysis of vaccine studies*. New York: Springer Science & Business Media.
6. Hanquet G., Valenciano M., Simondon F., Moren A. (2013) Vaccine effects and impact of vaccination programmes in post-licensure studies. *Vaccine* 31: 5634-5642.
