# Supplementary material for: Systematic literature review of the impact and effectiveness of monovalent meningococcal C conjugated vaccines when used in routine immunization programs
Source: BMC Public Health. 2020 Dec 9;20:1890. doi: 10.1186/s12889-020-09946-1 (PMC7724720; doi:10.1186/s12889-020-09946-1)
Supplement: Supplementary file 2 — Additional file 2: Supplementary figure 1. Vaccine effectiveness of MCCV in other countries. Supplementary Table 1. Summary characteristics of the studies included in the review. [file 12889_2020_9946_MOESM2_ESM.docx]

**Supplementary figure 1: Vaccine effectiveness of MCCV in other countries**


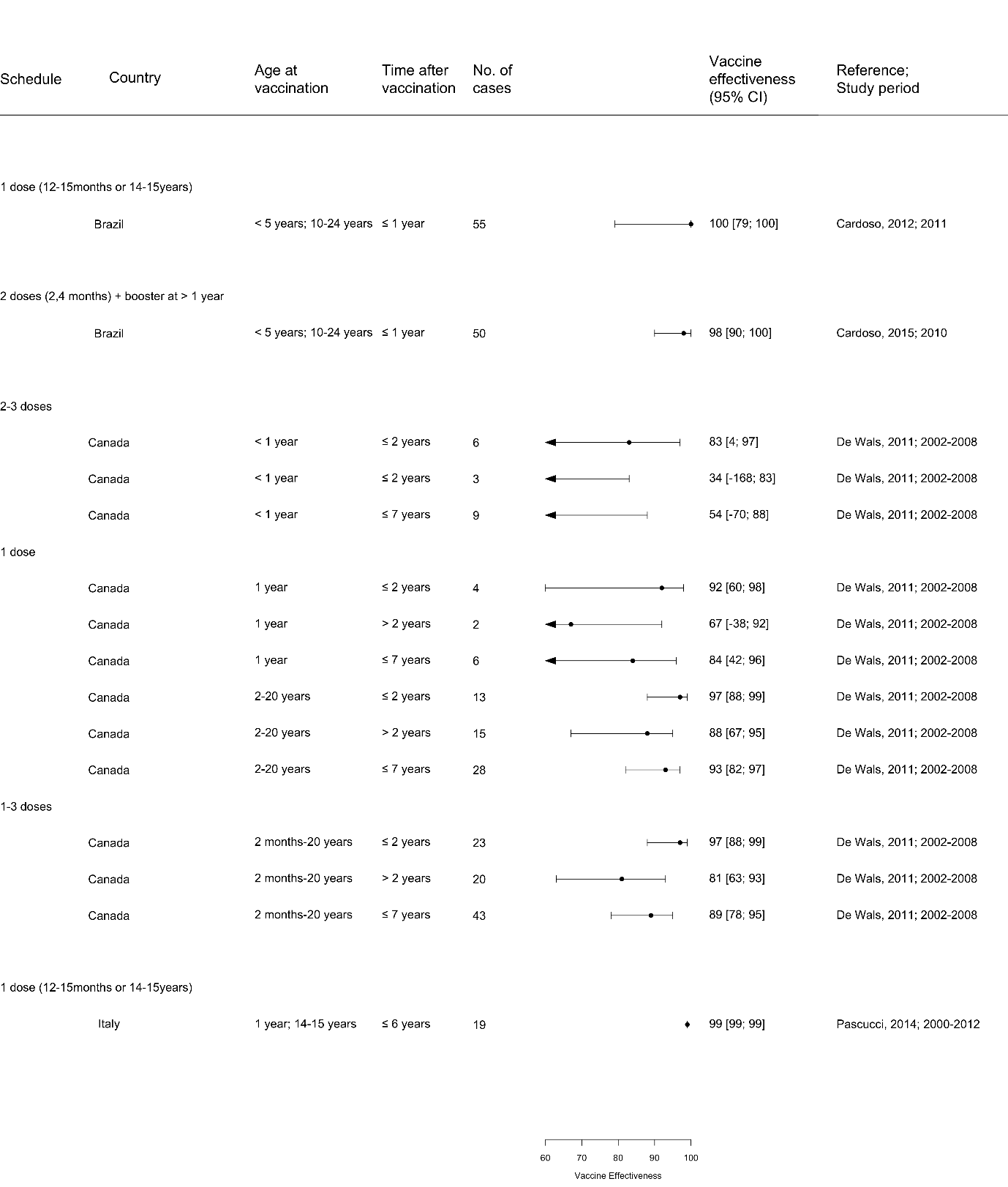


**Supplementary Table 1. Summary characteristics of the studies included in the review**

| **Reference, year of publication** | **Geographical location** | **Study design** | **Study period** | **Study population** |
| --- | --- | --- | --- | --- |
| **IMPACT STUDIES** | | | | |
| **Australia (MCCV introduced 2003)** | | | | |
| Chiu, 2010 [1] | Australia (National) | Surveillance | 1993-2007 | All ages |
| Lawrence, 2016 [2] | Australia (National) | Surveillance | 2000-2012 | All ages |
| **Belgium (MCCV introduced 2002)** | | | | |
| Mattheus, 2015 [3] | Belgium (National) | Surveillance | 1997-2012 | All ages |
| **Brazil (MCCV introduced 2010)** | | | | |
| Andrade, 2017 [4] | Brazil (National excluding Salvador; additional data for Sao Paulo only) | Ecological, interrupted time-series analysis | 2008-2014 | All ages |
| Cardoso, 2012 [5] | Brazil (Salvador City) | Surveillance | 2008-2011 | All ages |
| Tauil, 2014 [6] | Brazil (Federal District) | Surveillance | 2005-2011 | All ages |
| **Canada (MCCV introduction 2001-2005)** | | | | |
| Bettinger, 2009 [7] | Canada (National) | Surveillance | 2002-2006 | All ages |
| Kinlin, 2009 [8] | Canada (Ontario) | Surveillance | 2000-2006 | All ages |
| Siu, 2008 [9] | Canada (British Columbia) | Surveillance | 2003-2005 | All ages |
| Sadarangani, 2014 [10] | Canada (National; Quebec only; Alberta only; British Columbia only) | Surveillance | 2002-2012 | All ages |
| **Germany (MCCV introduction 2006)** | | | | |
| Hellenbrand, 2013 [11] | Germany (National) | Surveillance | 2002-2010 | All ages |
| **Italy (MCCV introduction 2005-2007)** | | | | |
| Stefanelli, 2009 [12] | Italy (National) | Surveillance | 2004-2007 | All ages |
| De Waure, 2016 [13] | Italy (National) | Surveillance | 1994-2012 | All ages |
| Neri, 2015 [14] | Italy (National) | Surveillance | 2008-2013 | All ages |
| Pascucci, 2014 [15] | Emilia Romagna | Surveillance | 2000-2012 | All ages |
| **Netherlands (MCCV introduction 2002)** | | | | |
| Bijlsma, 2014 [16] | National | Surveillance | 1998-2012 | All ages |
| Bijlsma, 2014 [17] | National | Surveillance | 1960-2012 | All ages |
| **Spain (MCCV introduction 2000)** | | | | |
| Cano, 2004 [18] | Spain (National) | Surveillance | 1999-2003 | All ages |
| Cruz Rojo, 2005 [19] | Spain (Andalusia) | Surveillance | 1997-2004 | All ages |
| Garrido, 2014 [20] | Spain (National) | Surveillance | 1997-2013 | All ages |
| Morales, 2016 [21] | Spain (Navarra) | Surveillance | 1995-2014 | All ages |
| Rego, 2011 [22] | Spain (Galicia) | Surveillance | 2004-2008 | All ages |
| Salleras, 2003 [23] | Spain (National) | Surveillance | 1997-2001 | All ages |
| Martinez, 2009 [24] | Spain (Catalonia) | Surveillance | 1997-2008 | All ages |
| **UK (MCCV introduction 1999)** | | | | |
| Balmer, 2002 [25] | England | Enhanced surveillance | 1999-2001 | <20 years |
| Miller, 2001 [26] | England and Wales | Surveillance | 1998-2001 | <18 years |
| Gray, 2006 [27] | England and Wales | Surveillance | 1993-2004 | All ages |
| Ramsay, 2003 [28] | England | Surveillance | 1998-2002 | <21 years |
| Stanton, 2011 [29] | Merseyside, England | Surveillance | 1997-2007 | 0-16 years |
| Trotter, 2002 [30] | England and Wales | Surveillance | 1998-2001 | All ages |
| **EFFECTIVENESS STUDIES** | | | | |
| **Brazil (MCCV introduction 2010)** | | | | |
| Cardoso, 2012 [5] | Salvador | Screening method | 2008-2011 | <5 years; 10-24 years |
| Cardoso, 2015 [31] | Salvador | Retrospective case-control | 2010-2010 | <5 years; 10-24 years |
| **Canada (MCCV introduction – Quebec – 2002)** | | | | |
| De Wals, 2011 [32] | Canada (Quebec) | Retrospective cohort | 1990-2008 | <20 years |
| **Italy (MCCV introduction 2006)** | | | | |
| Pascucci, 2014 [15] | Italy (Emilia-Romagna) | Screening method | 2000-2012 | <1 year; 15-16 years |
| **Spain (MCCV introduction 2000)** | | | | |
| Garrido, 2014 [20] | Spain (National) | Screening method | 1997-2013 | 2 months-20 years |
| Garrido, 2015 [33] | Spain (National) | Screening method | 2001-2013 | <20 years |
| Larrauri, 2005 [34] | Spain (National) | Screening method | 1999-2004 | <6 years |
| Morales, 2016 [21] | Spain (Navarra) | Screening method / indirect cohort | 2000 | <25 years |
| Salleras, 2003 [35] | Spain (Catalonia) | Retrospective cohort | 1997-2002 | <6 years |
| **UK (MCCV introduction 1999)** | | | | |
| Balmer, 2002 [25] | England | Screening method | 1999-2001 | 2-5 months; 1-17 years |
| Bose, 2003 [36] | 6 regions of England | Screening method and case-control | 1999-2001 | 15-19 years |
| Campbell, 2010 [37] | England | Screening method | 2000-2009 | 2 months-18 years |
| Miller, 2001 [26] | England | Screening method | 1999-2001 | 12-30 months; 15-17 years |
| Mooney, 2004 [38] | Scotland | Screening method | 1994-2003 | All ages |
| Ramsay, 2001 [39] | England | Screening method / retrospective cohort | 2000-2000 | 1-2 years; 15-17 years |
| Ramsay, 2003 [28] | England | Retrospective cohort | 1998-2002 | All ages |
| Trotter, 2004 [40] | England | Screening method | 2000-2004 | 2 months-18 years |

1. Chiu C, Dey A, Wang H, Menzies R, Deeks S, Mahajan D, et al. Vaccine preventable diseases in Australia, 2005 to 2007. Communicable diseases intelligence quarterly report. 2010;34 Supp:S1-167. PubMed PMID: 21416762.

2. Lawrence GL, Wang H, Lahra M, Booy R, Mc IP. Meningococcal disease epidemiology in Australia 10 years after implementation of a national conjugate meningococcal C immunization programme. Epidemiology and infection. 2016;144(11):2382-91. Epub 2016/04/21. doi: 10.1017/s0950268816000704. PubMed PMID: 27094814.

3. Mattheus W, Hanquet G, Collard JM, Vanhoof R, Bertrand S. Changes in Meningococcal Strains in the Era of a Serogroup C Vaccination Campaign: Trends and Evolution in Belgium during the Period 1997-2012. PloS one. 2015;10(10):e0139615. Epub 2015/10/02. doi: 10.1371/journal.pone.0139615. PubMed PMID: 26425857; PubMed Central PMCID: PMCPMC4591272.

4. Andrade AL, Minamisava R, Tomich LM, Lemos AP, Gorla MC, de Cunto Brandileone MC, et al. Impact of meningococcal C conjugate vaccination four years after introduction of routine childhood immunization in Brazil. Vaccine. 2017;35(16):2025-33. Epub 2017/03/21. doi: 10.1016/j.vaccine.2017.03.010. PubMed PMID: 28318769.

5. Cardoso CW, Pinto LL, Reis MG, Flannery B, Reis JN. Impact of vaccination during an epidemic of serogroup C meningococcal disease in Salvador, Brazil. Vaccine. 2012;30(37):5541-6. Epub 2012/07/04. doi: 10.1016/j.vaccine.2012.06.044. PubMed PMID: 22749604.

6. Tauil MdC, Carvalho CSRd, Vieira AC, Waldman EA. Meningococcal disease before and after the introduction of meningococcal serogroup C conjugate vaccine. Federal District, Brazil. Brazilian Journal of Infectious Diseases. 2014;18(4):379-86. PubMed PMID: rayyan-11608379.

7. Bettinger JA, Scheifele DW, Le Saux N, Halperin SA, Vaudry W, Tsang R, et al. The impact of childhood meningococcal serogroup C conjugate vaccine programs in Canada. Pediatr Infect Dis J. 2009;28(3):220-4. Epub 2009/02/12. doi: 10.1097/INF.0b013e31819040e7. PubMed PMID: 19209096.

8. Kinlin LM, Jamieson F, Brown EM, Brown S, Rawte P, Dolman S, et al. Rapid identification of herd effects with the introduction of serogroup C meningococcal conjugate vaccine in Ontario, Canada, 2000-2006. Vaccine. 2009;27(11):1735-40. Epub 2009/02/03. doi: 10.1016/j.vaccine.2009.01.026. PubMed PMID: 19186206.

9. Siu T, Tang W, Dawar M, Patrick DM. Impact of routine immunization using meningococcal C conjugate vaccine on invasive meningococcal disease in British Columbia. Canadian journal of public health = Revue canadienne de sante publique. 2008;99(5):380-2. Epub 2008/11/18. PubMed PMID: 19009920.

10. Sadarangani M, Scheifele DW, Halperin SA, Vaudry W, Le Saux N, Tsang R, et al. The impact of the meningococcal serogroup C conjugate vaccine in Canada between 2002 and 2012. Clin Infect Dis. 2014;59(9):1208-15. Epub 2014/07/30. doi: 10.1093/cid/ciu597. PubMed PMID: 25069868.

11. Hellenbrand W, Elias J, Wichmann O, Dehnert M, Frosch M, Vogel U. Epidemiology of invasive meningococcal disease in Germany, 2002-2010, and impact of vaccination with meningococcal C conjugate vaccine. The Journal of infection. 2013;66(1):48-56. Epub 2012/10/10. doi: 10.1016/j.jinf.2012.09.008. PubMed PMID: 23043893.

12. Stefanelli P, Fazio C, Sofia T, Neri A, Mastrantonio P. Serogroup C meningococci in Italy in the era of conjugate menC vaccination. BMC infectious diseases. 2009;9:135. Epub 2009/08/25. doi: 10.1186/1471-2334-9-135. PubMed PMID: 19698137; PubMed Central PMCID: PMCPMC2739211.

13. de Waure C, Miglietta A, Nedovic D, Mereu G, Ricciardi W. Reduction in Neisseria meningitidis infection in Italy after Meningococcal C conjugate vaccine introduction: A time trend analysis of 1994-2012 series. Human vaccines & immunotherapeutics. 2016;12(2):467-73. Epub 2015/08/27. doi: 10.1080/21645515.2015.1078951. PubMed PMID: 26308192; PubMed Central PMCID: PMCPMC5049743.

14. Neri A, Pezzotti P, Fazio C, Vacca P, D'Ancona FP, Caporali MG, et al. Epidemiological and Molecular Characterization of Invasive Meningococcal Disease in Italy, 2008/09-2012/13. PloS one. 2015;10(10):e0139376. Epub 2015/10/09. doi: 10.1371/journal.pone.0139376. PubMed PMID: 26445461; PubMed Central PMCID: PMCPMC4596568.

15. Pascucci MG, Di Gregori V, Frasca G, Rucci P, Finarelli AC, Moschella L, et al. Impact of meningococcal C conjugate vaccination campaign in Emilia-Romagna, Italy. Human vaccines & immunotherapeutics. 2014;10(3):671-6. Epub 2014/01/05. PubMed PMID: 24384537; PubMed Central PMCID: PMCPMC4130270.

16. Bijlsma MW, Brouwer MC, Spanjaard L, van de Beek D, van der Ende A. A decade of herd protection after introduction of meningococcal serogroup C conjugate vaccination. Clinical infectious diseases : an official publication of the Infectious Diseases Society of America. 2014;59(9):1216-21. Epub 2014/07/30. doi: 10.1093/cid/ciu601. PubMed PMID: 25069869.

17. Bijlsma MW, Bekker V, Brouwer MC, Spanjaard L, van de Beek D, van der Ende A. Epidemiology of invasive meningococcal disease in the Netherlands, 1960-2012: an analysis of national surveillance data. The Lancet Infectious diseases. 2014;14(9):805-12. Epub 2014/08/12. doi: 10.1016/s1473-3099(14)70806-0. PubMed PMID: 25104306.

18. Cano R, Larrauri A, Mateo S, Alcala B, Salcedo C, Vazquez JA. Impact of the meningococcal C conjugate vaccine in Spain: an epidemiological and microbiological decision. Euro surveillance : bulletin Europeen sur les maladies transmissibles = European communicable disease bulletin. 2004;9(7):11-5. Epub 2004/08/20. PubMed PMID: 15318008.

19. Cruz Rojo C, García Gil C, Nieto Vera J, Monroy Morcillo A. Enfermedad meningocócica e impacto de la vacunación sistemática con la vacuna conjugada antimeningocócia C en un área sanitaria de Andalucía. Revista Española de Salud Pública. 2005;79(6):655-63. PubMed PMID: rayyan-11608359.

20. Garrido-Estepa M, Leon-Gomez I, Herruzo R, Cano R. Changes in meningococcal C epidemiology and vaccine effectiveness after vaccine introduction and schedule modification. Vaccine. 2014;32(22):2604-9. doi: 10.1016/j.vaccine.2014.03.010. PubMed PMID: 24662700.

21. Morales D, Garcia-Cenoz M, Moreno L, Bernaola E, Barricarte A, Castilla J. [Meningococcal C conjugate vaccine: Impact of a vaccination program and long-term effectiveness in Navarra, Spain, 2000-2014]. Enfermedades infecciosas y microbiologia clinica. 2016;34(10):639-44. doi: 10.1016/j.eimc.2015.11.020. PubMed PMID: 26778101.

22. Rego Romero E, Nartallo Penas V, Taboada Rodriguez JA, Malvar Pintos A, Hervada Vidal X, Lopez Pimentel MJ. Implementation and impact of a meningococcal C conjugate vaccination program in 13- to 25-year-old individuals in Galicia, Spain. Zeitschrift fur Gesundheitswissenschaften = Journal of public health. 2011;19(5):409-15. Epub 2011/10/01. doi: 10.1007/s10389-011-0403-9. PubMed PMID: 21957332; PubMed Central PMCID: PMCPMC3172415.

23. Salleras L, Dominguez A, Cardenosa N. Impact of mass vaccination with polysaccharide conjugate vaccine against serogroup C meningococcal disease in Spain. Vaccine. 2003;21(7-8):725-8. Epub 2003/01/18. PubMed PMID: 12531349.

24. Martínez AI, Domínguez Á, Oviedo M, Minguell S, Jansà JM, Codina G, et al. Epidemiología de la enfermedad meningocócica en Cataluña antes y después de la vacunación frente al serogrupo C. Revista Española de Salud Pública. 2009;83(5):725-35. PubMed PMID: rayyan-11608368.

25. Balmer P, Borrow R, Miller E. Impact of meningococcal C conjugate vaccine in the UK. Journal of medical microbiology. 2002;51(9):717-22. Epub 2002/10/03. doi: 10.1099/0022-1317-51-9-717. PubMed PMID: 12358061.

26. Miller E, Salisbury D, Ramsay M. Planning, registration, and implementation of an immunisation campaign against meningococcal serogroup C disease in the UK: a success story. Vaccine. 2001;20 Suppl 1:S58-67. PubMed PMID: 11587814.

27. Gray SJ, Trotter CL, Ramsay ME, Guiver M, Fox AJ, Borrow R, et al. Epidemiology of meningococcal disease in England and Wales 1993/94 to 2003/04: contribution and experiences of the Meningococcal Reference Unit. Journal of medical microbiology. 2006;55(Pt 7):887-96. Epub 2006/06/15. doi: 10.1099/jmm.0.46288-0. PubMed PMID: 16772416.

28. Ramsay ME, Andrews NJ, Trotter CL, Kaczmarski EB, Miller E. Herd immunity from meningococcal serogroup C conjugate vaccination in England: database analysis. BMJ (Clinical research ed). 2003;326(7385):365-6. Epub 2003/02/15. PubMed PMID: 12586669; PubMed Central PMCID: PMCPMC148893.

29. Stanton MC, Taylor-Robinson D, Harris D, Paize F, Makwana N, Hackett SJ, et al. Meningococcal disease in children in Merseyside, England: a 31 year descriptive study. PloS one. 2011;6(10):e25957. Epub 2011/10/22. doi: 10.1371/journal.pone.0025957. PubMed PMID: 22016791; PubMed Central PMCID: PMCPMC3189236.

30. Trotter CL, Ramsay ME, Kaczmarski EB. Meningococcal serogroup C conjugate vaccination in England and Wales: coverage and initial impact of the campaign. Communicable disease and public health. 2002;5(3):220-5. Epub 2002/11/19. PubMed PMID: 12434692.

31. Cardoso CW, Ribeiro GS, Reis MG, Flannery B, Reis JN. Effectiveness of meningococcal C conjugate vaccine in Salvador, Brazil: a case-control study. PloS one. 2015;10(4):e0123734. Epub 2015/04/16. doi: 10.1371/journal.pone.0123734. PubMed PMID: 25874777; PubMed Central PMCID: PMCPMC4395143.

32. De Wals P, Deceuninck G, Lefebvre B, Boulianne N, De Serres G. Effectiveness of serogroup C meningococcal conjugate vaccine: a 7-year follow-up in Quebec, Canada. The Pediatric infectious disease journal. 2011;30(7):566-9. Epub 2011/02/18. doi: 10.1097/INF.0b013e31820e8638. PubMed PMID: 21326136.

33. Garrido-Estepa M, Nunez OG, Leon-Gomez I, Cano R, Herruzo R. Meningococcal C conjugate age-dependant long-term loss of effectiveness. Vaccine. 2015;33(19):2221-7. Epub 2015/03/31. doi: 10.1016/j.vaccine.2015.03.044. PubMed PMID: 25819711.

34. Larrauri A, Cano R, Garcia M, Mateo S. Impact and effectiveness of meningococcal C conjugate vaccine following its introduction in Spain. Vaccine. 2005;23(32):4097-100. Epub 2005/05/24. doi: 10.1016/j.vaccine.2005.03.045. PubMed PMID: 15908059.

35. Salleras L, Dominguez A, Cardenosa N. Dramatic decline of serogroup C meningococcal disease in Catalonia (Spain) after a mass vaccination campaign with meningococcal C conjugated vaccine. Vaccine. 2003;21(7-8):729-33. Epub 2003/01/18. PubMed PMID: 12531350.

36. Bose A, Coen P, Tully J, Viner R, Booy R. Effectiveness of meningococcal C conjugate vaccine in teenagers in England. Lancet (London, England). 2003;361(9358):675-6. Epub 2003/02/28. doi: 10.1016/s0140-6736(03)12563-9. PubMed PMID: 12606181.

37. Campbell H, Andrews N, Borrow R, Trotter C, Miller E. Updated postlicensure surveillance of the meningococcal C conjugate vaccine in England and Wales: effectiveness, validation of serological correlates of protection, and modeling predictions of the duration of herd immunity. Clinical and vaccine immunology : CVI. 2010;17(5):840-7. doi: 10.1128/CVI.00529-09. PubMed PMID: 20219881; PubMed Central PMCID: PMCPMC2863391.

38. Mooney JD, Christie P, Robertson C, Clarke SC. The impact of meningococcal serogroup C conjugate vaccine in Scotland. Clinical infectious diseases : an official publication of the Infectious Diseases Society of America. 2004;39(3):349-56. Epub 2004/08/13. doi: 10.1086/421947. PubMed PMID: 15307001.

39. Ramsay ME, Andrews N, Kaczmarski EB, Miller E. Efficacy of meningococcal serogroup C conjugate vaccine in teenagers and toddlers in England. Lancet (London, England). 2001;357(9251):195-6. doi: 10.1016/S0140-6736(00)03594-7. PubMed PMID: 11213098.

40. Trotter CL, Andrews NJ, Kaczmarski EB, Miller E, Ramsay ME. Effectiveness of meningococcal serogroup C conjugate vaccine 4 years after introduction. Lancet (London, England). 2004;364(9431):365-7. doi: 10.1016/S0140-6736(04)16725-1. PubMed PMID: 15276396.
